# Overexpression of *PSY1* increases fruit skin and flesh carotenoid content and reveals associated transcription factors in apple (*Malus* × *domestica*)

**DOI:** 10.3389/fpls.2022.967143

**Published:** 2022-09-15

**Authors:** Charles Ampomah-Dwamena, Sumathi Tomes, Amali H. Thrimawithana, Caitlin Elborough, Nitisha Bhargava, Ria Rebstock, Paul Sutherland, Hilary Ireland, Andrew C. Allan, Richard V. Espley

**Affiliations:** ^1^The New Zealand Institute for Plant and Food Research Ltd., Auckland, New Zealand; ^2^BioLumic Limited, Palmerston North, New Zealand

**Keywords:** carotenoid biosynthesis, transgenic apple, transcriptome, plastids, gene expression, phytoene synthase, transcription factors

## Abstract

Knowledge of the transcriptional regulation of the carotenoid metabolic pathway is still emerging and here, we have misexpressed a key biosynthetic gene in apple to highlight potential transcriptional regulators of this pathway. We overexpressed phytoene synthase (*PSY1*), which controls the key rate-limiting biosynthetic step, in apple and analyzed its effects in transgenic fruit skin and flesh using two approaches. Firstly, the effects of PSY overexpression on carotenoid accumulation and gene expression was assessed in fruit at different development stages. Secondly, the effect of light exclusion on PSY1-induced fruit carotenoid accumulation was examined. PSY1 overexpression increased carotenoid content in transgenic fruit skin and flesh, with beta-carotene being the most prevalent carotenoid compound. Light exclusion by fruit bagging reduced carotenoid content overall, but carotenoid content was still higher in bagged PSY fruit than in bagged controls. In tissues overexpressing *PSY1*, plastids showed accelerated chloroplast to chromoplast transition as well as high fluorescence intensity, consistent with increased number of chromoplasts and carotenoid accumulation. Surprisingly, the expression of other carotenoid pathway genes was elevated in PSY fruit, suggesting a feed-forward regulation of carotenogenesis when this enzyme step is mis-expressed. Transcriptome profiling of fruit flesh identified differentially expressed transcription factors (TFs) that also were co-expressed with carotenoid pathway genes. A comparison of differentially expressed genes from both the developmental series and light exclusion  treatment revealed six candidate TFs exhibiting strong correlation with carotenoid accumulation. This combination of physiological, transcriptomic and metabolite data sheds new light on plant carotenogenesis and TFs that may play a role in regulating apple carotenoid biosynthesis.

## Introduction

Carotenoids play essential roles in photosynthesis as well as conferring coloration in flower and fruit tissues for pollination, seed dispersal and increased consumer appeal (Zhu et al., 2010). These pigments are also noted for their health associations, notably as precursors of vitamin A and as scavengers of free oxygen species (Weber and Grune, 2012; Eggersdorfer and Wyss, 2018; Rodríguez-Concepcíon et al., 2018). Despite the association of carotenoid pigments with health benefits, and their presence in apple skin, most commercial apple cultivars have little or no pigmentation in the fruit flesh at maturity, making the regulation of secondary metabolic pathways controlling these pigments in the fruit flesh an important area of research (Ampomah-Dwamena et al., 2012; Delgado-Pelayo et al., 2014). Apple fruit are widely consumed globally and increasing phytochemical content, such as carotenoids, could well improve their nutritional and commercial value. Although the long juvenile period in apple presents a significant challenge to breeding, knowledge of the carotenoid metabolic pathway and its regulation can contribute to accelerated breeding of a higher-carotenoid apple.

Carotenoid accumulation in tissues is a balance between biosynthesis and metabolite breakdown, controlled by the upstream pathway biosynthetic enzymes (regulating flux) and downstream breakdown enzymes (metabolite turnover), respectively (Brandi et al., 2011; Rodríguez-Concepcíon et al., 2018). The carotenoid enzymatic pathway is situated in the plastids and metabolically connected to the methylerythritol 4-phosphate (MEP) pathway, which generates the C_20_ compound geranylgeranyl pyrophosphate (GGPP), and which also is a common precursor for gibberellins, tocopherols, and chlorophylls (Okada et al., 2000). In the carotenoid pathway, GGPP is a substrate for the first committed enzymatic condensation of two GGPP molecules to give the colorless phytoene (Nisar et al., 2015; You et al., 2020). This step, catalyzed by phytoene synthase (PSY), is a well-regulated rate-limiting step in many plant species (Welsch et al., 2010) and controls the carotenoid pathway flux, as indicated by the amount of phytoene that accumulates in the presence of an attenuated phytoene desaturase (PDS) (Schaub et al., 2018). PDS catalyzes a two-step desaturation of phytoene into phytofluene and then (tri-*cis*) zeta-carotene, which becomes a substrate for zeta-carotene isomerase (Z-ISO) for conversion to di-*cis* zeta-carotene (Chen et al., 2010; Beltrán et al., 2015). Subsequent desaturation and isomerization by zeta-carotene desaturase (ZDS) and carotene isomerase (CRTISO), respectively, result in lycopene, providing the substrate for the lycopene cyclases to produce alpha-carotene and beta-carotene in a bifurcated pathway step (Isaacson et al., 2004). The conversion of alpha-carotene to lutein, and beta-carotene to zeaxanthin, is through the actions of carotene hydroxylases (Kim and DellaPenna, 2006; Quinlan et al., 2012; Niu et al., 2020).

Carotenoid content in tissues can thus be altered by manipulating the expression of rate-limiting steps such as PSY to intensify pathway flux (‘Push’ strategy) or by reducing downstream enzymatic turnover of accumulated compounds using a ‘Block’ approach (Diretto et al., 2010; Zeng et al., 2015). In the latter, carotenoid biosynthetic enzymes or degradation enzymes such as carotenoid cleavage dioxygenases (CCDs) or nine *cis* epoxy-carotenoid dioxygenases (NCEDs) can be downregulated to increase carotenoid content (Ko et al., 2018). Although the ‘Push’ strategy has been widely utilized to increase carotenoid content in various tissues (Ducreux et al., 2005; Zhou et al., 2022), its success depends on the efficiency of subsequent pathway steps and having the appropriate storage subcellular compartments in these tissues. For instance, the increased carotenoid content in tomato fruit as a result of *PSY1* overexpression was dependent on fruit stage, feed-forward effect on pathway and abundance of chromoplasts required for carotenoid storage (Fraser et al., 2007). Six apple PSY genes are present in the ‘Golden Delicious’ genome sequence and their homologs have been characterized in other apple cultivars (Velasco et al., 2010; Ampomah-Dwamena et al., 2012, 2015; Zhang et al., 2018; Cerda et al., 2020). These studies have mainly focused on gene expression analysis and functional characterization in heterologous systems. However, increased apple *PSY* gene expression has indirectly been associated with increased fruit carotenoid content. The overexpression in apple of the Arabidopsis gene AtDXR, which is an enzyme in the upstream MEP pathway, increased fruit carotenoid content with associated increased expression of apple *PSY* genes (Arcos et al., 2020). More recently, the overexpression of *MdAP2-34* transcription factor in apple also increased fruit carotenoid content and upregulated expression of *MdPSY2* (Dang et al., 2021). This positive relationship between apple PSY gene expression and fruit carotenoid content indicates a ‘push’ strategy can be utilized to boost apple carotenoid concentration.

Plastid differentiation into chromoplasts offers another approach (expanding capacity) to increase carotenoid accumulation. The gain-of-function *Orange* (*OR*) gene mutation in cauliflower and subsequently the transgenic overexpression of *ORs* has increased carotenoid content in many plants (Li and Van Eck, 2007; Park et al., 2016). The OR protein, in addition to promoting plastid differentiation, post-transcriptionally regulates PSY protein content and activity, while the golden SNP *OR^HIS^* mutation has been linked to downregulation of beta-carotene hydroxylase expression, resulting in increased beta-carotene content (Tzuri et al., 2015; Zhou et al., 2015; Park et al., 2016; Chayut et al., 2017). These observations of OR functions provide a snapshot of the linkages between the different mechanisms (both translational and post-translational) controlling carotenoid accumulation and further highlight the complex regulation of this metabolic pathway.

The carotenoid pathway is subject to various degrees of regulation, which affect carotenoid biosynthesis, and determine the accumulation in both photosynthetic and non-photosynthetic tissues (Sun and Li, 2020). Whereas transcriptional regulation of the pathway has received significant attention and led to the identification of many carotenoid transcription factors (TFs; Stanley and Yuan, 2019), post-transcriptional and post-translational mechanisms also exert some control on this process. Alternative splicing of carotenoid genes presents a way to exert control of the pathway, especially with pivotal genes such as *PSY*. Alternative splicing of Arabidopsis PSY has been linked with different translation efficiency, owing to the variation in their 5’ untranslated regions, and different regulatory modules that respond to either carotenoid flux or abiotic stress (Álvarez et al., 2016). In crocus, spliced variants of *CsPSY1b* exhibited differential expression, with the presence of the intron-containing variant associated with tissues having reduced transcript levels (Ahrazem et al., 2019). Alternative splicing of wheat PSY-A1 resulted in four mRNA variants that affected the abundance of the wild-type transcript, which was the only one that produced an enzymatically active protein (Howitt et al., 2009). In tomato, alternative *trans*-splicing of *PSY1* resulted in a longer chimeric variant that was responsible for the yellow flesh *yft2* fruit phenotype (Chen et al., 2019). Additional post-translational control mechanisms play a role in altering the activity of carotenoid enzymes. For instance the abundance of PSY in the plastids is regulated by the OR protein with the amount of PSY protein significantly reduced in the *or* mutants of Arabidopsis and melon (Zhou et al., 2015; Chayut et al., 2017). While the mechanism for this OR-induced stability is not fully established, the holdase chaperone activity of OR plays a role by preventing PSY protein aggregation and subsequent degradation (Park et al., 2016). The activity of ATP-dependent serine type caseinolytic (Clp) protease, which targets protein for degradation, regulates the protein content and activity of PSY and other downstream pathway enzymes (Welsch et al., 2018). The silencing of the Clp protease activity in tomato resulted in increased expression of the *OR* gene and enhanced beta-carotene accumulation in fruit, highlighting the link between the holdase and protease activities of OR and Clp, respectively (D’Andrea et al., 2018; D’Andrea and Rodriguez-Concepcion, 2019).

Transcriptional regulation of the carotenoid pathway appears to vary among plant species, with a diversity of TFs reported to control the pathway. We previously identified a kiwifruit *MYB7* gene whose expression induced key pathway genes, implicating it has a role in carotenogenesis (Ampomah-Dwamena et al., 2019). MYB TFs such as CrMYB68 in citrus function as negative regulators of CrBCH2 and CrNCEDs (Zhu F. et al., 2017), whilst RCP1, another R2R3 MYB belonging to subgroup 21, is a positive regulator of carotenoid accumulation in *Erythranthe lewisii* flowers (Sagawa et al., 2016). Similarly, *Medicago truncatula* White Petal 1 protein (a homolog of AtMYB113 and in a different subgroup from RCP1) also transcriptionally activates carotenoid biosynthetic genes through its interaction with other proteins (Meng et al., 2019). Reports of potential transcriptional regulators of the carotenoid pathway belonging to TF classes, such as NACs, MADS, ERF, and bHLH, have been reviewed recently, highlighting that carotenoid pathway regulation may have evolved independently in different plant species (Stanley and Yuan, 2019). This apparent lack of conserved TF activity among plant species and the lack of pathway mutants makes it particularly important to adapt new strategies for identifying carotenoid TFs in discrete species. It is possible that the perturbations caused by transgene expression, for instance, could uncover gene network relationships controlling the observed phenotype. The overexpression of *PSY* in tomato, which increased carotenoid accumulation, also elicited various molecular and metabolic responses to uncover carotenoid pathway regulation in this species (Fraser et al., 2007). The molecular changes resulting from such induced expression could help outline the transcriptional regulatory networks involved in carotenogenesis and potentially highlight candidate TF genes involved in this metabolic process. In this study, we set out to understand the mechanisms controlling the apple carotenoid biosynthetic pathway by overexpressing the rate-limiting *phytoene synthase 1* (*PSY1*). Analyses of physiological, metabolic and transcriptional changes shed new light on the regulation of carotenoid metabolic pathway in this species.

## Results

### *PSY1* expression increased fruit carotenoid content

**FIGURE 1:**
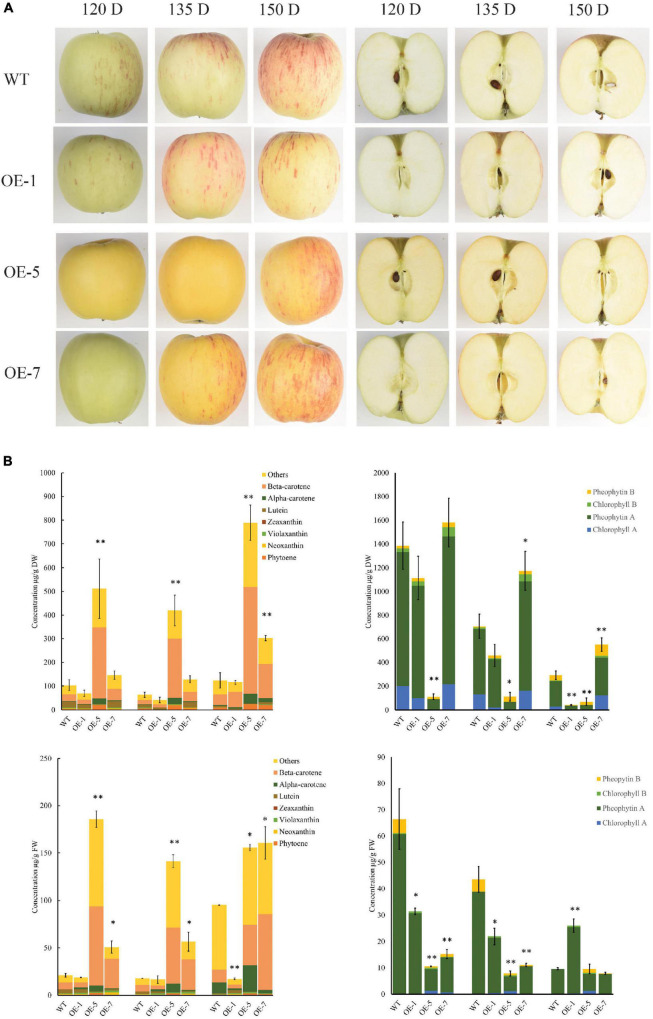
PSY1 expression increased carotenoid accumulation in ‘Royal Gala’ apple fruit. **(A)** Fruit of WT and PSY transgenic lines (OE-1, OE-5, and OE-7) at different developmental stages. **(B)** Bar graphs of carotenoid content measured by HPLC as beta-carotene equivalents (left column) and chlorophyll (right column) in fruit skin (top panel) and fruit flesh (bottom panel) in wild type and transgenic lines OE-1, OE-5, and OE-7. The error bars represent the standard errors of the mean of three biological replicates, with each replicate a pool of 5–7 fruit. Bar graph with asterisk show significant difference from WT at the same fruit stage using Dunnett’s test (*P < 0.05; **P < 0.01).

We previously described six *PSY* genes present in the ‘Golden Delicious’ apple genome and characterized their homologs in ‘Royal Gala’ (Velasco et al., 2010; Ampomah-Dwamena et al., 2015). We selected *PSY1* (located on chromosome 17), whose expression strongly correlated with carotenoid accumulation in apple fruit skin and flesh, and expressed it constitutively using the CMV:35S promoter in ‘Royal Gala’ apple. To test if PSY1 was capable of creating enhanced pathway flux, we first generated transformed apple callus, with and without norflurazon (NFZ), an inhibitor of the next pathway step (PDS) and then measured phytoene content (Schaub et al., 2018; Koschmieder and Welsch, 2020). Without NFZ, the phytoene content in the PSY calli was up to 15-fold higher than in WT (Supplementary Figure S1). In the presence of NFZ, phytoene content increased a further two- to three-fold in PSY calli and was approximately six times higher than in WT callus on the same treatment. Other downstream compounds such as beta-carotene, alpha-carotene, lutein and zeaxanthin that accumulated in these calli, showed little to no change with NFZ treatment (Supplementary Figure S1). Next, we generated stably transformed *PSY1* overexpressed (OE) transgenic plants, with wild type (WT) as control, for analyses. During tissue culture regeneration and growth in soil, vegetative tissues of transgenic plants showed normal phenotype. However, during fruit development, the transgenic plants displayed a deep yellow fruit color phenotype (Figure 1A).

Carotenoid accumulation was analyzed in the fruit skin and flesh tissues of three transgenic PSY lines with sufficient fruit numbers on the tree (OE-1, OE-5, and OE-7), and WT as control at four developmental stages: 90, 120, 135, and 150 days after pollination (D).

The PSY fruit, at the early stages (90 and 120 D), had green skin and white flesh color and, in the later stages (135 and 150 D), both flesh and skin developed strong yellow pigmentation (Figure 1A). The control wild-type (WT) fruit, in contrast, developed red streaks of anthocyanin pigments on a green background skin at 120 D, as expected. After this stage, the fruit skin began to de-green to reveal a yellowish background skin color. The WT fruit flesh color was whitish during the early fruit stages and acquired a creamy color when fruit were at maturity (150 D).

High-performance liquid chromatography (HPLC) analysis of carotenoid content and composition showed that beta-carotene was the dominant compound accumulating in both skin and flesh tissues, with the content increasing with fruit development. We also observed increased amounts of esterified compounds in both PSY and WT fruit at 135 and 150 D. Carotenoid content in OE-1 fruit did not increase compared with WT at all the four fruit stages we examined while, a three-fold to eight-fold increase in total carotenoid content (TCC) was observed in OE-5 and OE-7 fruit skin. TCC in fruit flesh of WT, OE-5 and OE-7 were 21.2 ± 1.93, 185.8 ± 8.7, 50.9 ± 6.5 μg/g FW at 120 D; 17.9 ± 0.1, 141.3 ± 6.9, 56.6 ± 10.0 μg/g FW at 135 D and 95.3 ± 0.4, 155.9 ± 3.4, 160.8 ± 17.0 μg/g FW at 150 D, respectively (Figure 1B). TCC in OE-5 and OE-7 flesh thus increased by 8.7, 2.4-fold at 120 D; 7.9, 3.2-fold at 135 D; and by 1.6, 1.7-fold at 150 D, respectively when compared with the WT flesh. The concentration of beta-carotene, the predominant compound in these tissues, increased by 3.2-fold in OE-5 (42.9 ± 12.6 μg/g FW) and 5.9-fold in OE-7 (79.8 ± 4.5 μg/g FW) compared with WT (13.3 ± 3.6 μg/g FW) at 150 D. This represented 27% (OE-5) and 50% (OE-7) of total carotenoids, compared with 14% for WT at that stage. In contrast, chlorophyll in fruit skin or flesh decreased as the fruit developed, with no clear difference between the PSY lines and WT fruit. Total chlorophyll content in WT flesh was higher than in PSY fruit at most of the stages we monitored (Figure 1B).

### Plastid changes in *PSY* transgenic fruit

**FIGURE 2:**
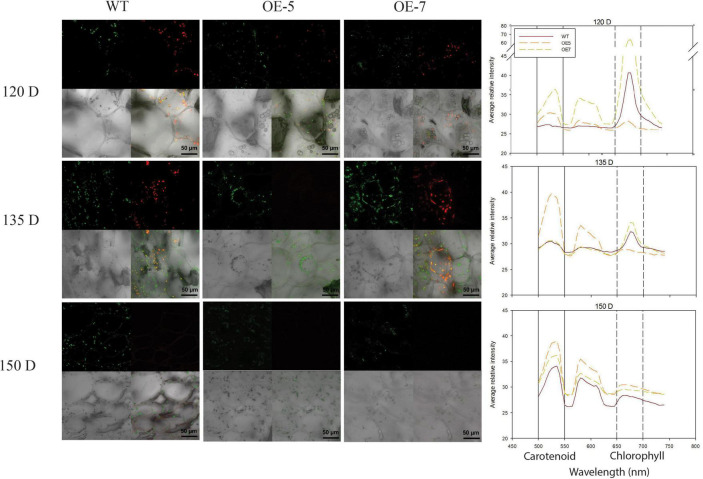
Visualization of chlorophyll and carotenoid autofluorescence in wild type (WT) and PSY ‘Royal Gala’ fruit. Fresh fruit tissues, at 120, 135, and 150 D, were analyzed by confocal microscopy to show plastids containing chlorophyll (red), carotenoid (green) and both pigments (yellow). Graph represents fluorescence emission spectra of WT and PSY transgenic tissue. Fluorescence emission between 500 and 600 nm represents carotenoids and 650–750, chlorophyll pigments.

Plastid transition in fruit was examined using confocal microscopy analysis of WT, OE-5, and OE-7 fruit at 120, 135, and 150 D. The chloroplasts, containing chlorophyll pigments, emitted red fluorescence under excitation, while the chromoplasts emitted green fluorescence. At 120 D, the WT fruit contained predominantly chloroplasts, which emitted red fluorescence, whilst in the two PSY lines at the same stage, there was a mixed population of chloroplasts and chromoplasts (green fluorescence), suggesting a hastened plastid transition. At 135 D, the WT had both plastid types in almost equal abundance, while mostly chromoplasts were present in the PSY fruit. At 150 D, which coincided with fruit ripening, mostly chromoplasts were observed in both PSY and WT tissues; however, in the PSY fruit these plastids were in abundance compared with their sparse distribution in WT fruit (Figure 2). The fluorescence emission spectra, a measure of carotenoid (550–650 nm) and chlorophyll (650–700 nm) content (Kilcrease et al., 2013; D’Andrea et al., 2014), further confirmed the increased plastid capacity in PSY fruit. Both the 120 D WT and PSY fruit showed a chlorophyll peak, but the PSY fruit showed additional carotenoid peaks, confirming early accumulation of carotenoids in the transgenic fruit. In both 135 D and 150 D stages, higher carotenoid fluorescent intensity was observed within PSY fruit than in the WT.

### Carotenoid gene expression in transgenic fruit

**FIGURE 3:**
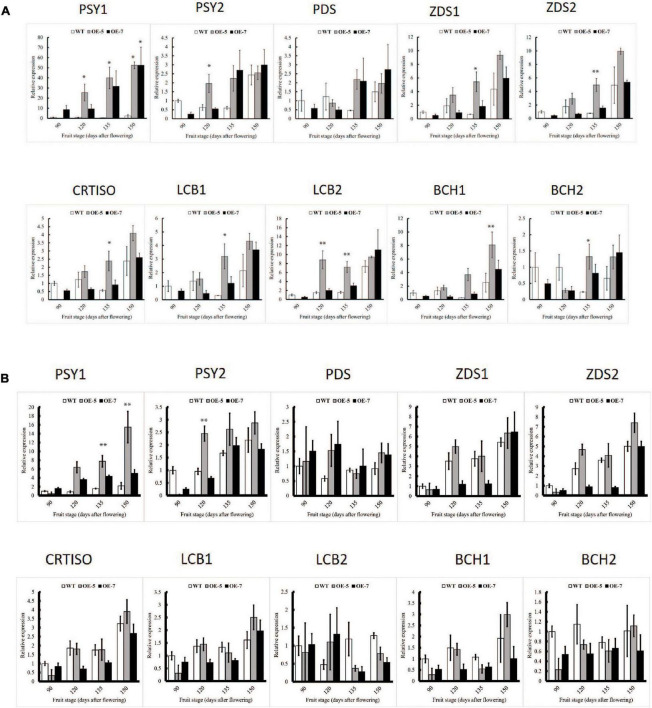
Comparison of carotenoid gene expression in wild-type (WT) and PSY ‘Royal Gala’ apple lines, OE-5 and OE-7, as determined by qPCR, relative to expression of housekeeping genes (*MdActin* and *MdEF1A*) in fruit skin **(A)** and fruit flesh **(B)**. Bars represent means and SE of four biological replicates. PSY, phytoene synthase; PDS, phytoene desaturase; ZDS, zeta carotene desaturase; CRTISO, carotene isomerase; LCB, lycopene beta-cyclase; BCH, beta-carotene hydroxylase. Bar graph with asterisk shows significant difference from WT at the same fruit stage using Dunnett’s test (*P < 0.05; **P < 0.01).

To understand the molecular mechanism underlying the increased carotenoid accumulation in PSY fruit, the relative transcript levels of carotenoid genes in fruit skin and flesh tissues from the OE-5, OE-7, and WT lines were assessed by real-time quantitative PCR (qPCR). *PSY1* expression in the transgenic lines was high (Figure 3A) and increased with development due to the cumulative contribution of both the endogenous gene and the 35S-driven transgene expression. In fruit skin, the expression of genes including *PSY2*, *ZDS1*, *ZDS2 CRTISO*, *LCB2* and *BCH1*, increased in the transgenic PSY lines compared with the WT and this was consistent across all developmental stages examined. Other genes however, including *PDS*, *ZISO*, *LCB1*, *BCH2* and *ZEP2*, showed higher expression in the WT fruit skin than in PSY lines at the 90 D and 120 D stages, but the trend was reversed at 135 and 150 D stages, with elevated expression in PSY lines. *LCYE* expression was higher in WT fruit than *PSY* at all four stages, while *ECH* showed higher expression in PSY fruit only at the 135 D stage (Figure 3A and Supplementary Figure S2). There was a similar trend for carotenoid gene expression in fruit flesh tissues, where transcript levels increased in the PSY fruit compared with the WT control, with the exception of the *LCB2* and *BCH2* genes, which showed higher transcript levels in the WT than in the PSY lines. The expression of *PDS, ZDS1*, *ZDS2*, *CRTISO* and *LCB1* was higher in the PSY lines than in the WT across all the fruit stages. In contrast, expression of *BCH1* and *BCH2* in PSY fruit reduced, compared with the WT, in the first three fruit stages but then increased at the 150 D stage (Figure 3B). Overall, PSY overexpression resulted in the upregulation of carotenoid pathway genes acting upstream of beta-carotene, and downregulation of genes downstream of beta-carotene. It appears that there is a coordinated regulatory response occurring in association with the mis-expression of PSY.

### Transcriptomic analysis identifies differentially expressed genes associated with *PSY1* expression

Transcriptional changes associated with *PSY1*-induced carotenoid accumulation in apple fruit flesh were characterized by transcriptome sequencing of OE-7, which showed a more consistent fruit carotenoid accumulation pattern, and WT at four stages (90, 120, 135, 150 D). A total sequence output of 180 GB from paired-end sequencing of 24 samples yielded an average of 25 million map-able reads per sample. Sequence reads were mapped to the ‘Golden Delicious’ double haploid (GDDH) genome (Daccord et al., 2017) to identify differentially expressed genes (DEGs), where the percentage of uniquely mapped reads was over 94%. Principal component analysis (PCA) of expression data showed that PC1 and PC2 explained 49% and 39% of variance, respectively, grouping samples according to treatment and fruit developmental stages (Supplementary Figure S3). For an overview of expressed carotenoid pathway genes in fruit, we analyzed reads from PSY fruit and WT that mapped to 54 gene models identified from the GDDH genome (Supplementary Table S2). Carotenoid genes that showed more than 1.5-fold increase (*p* < 0.05) in at least two PSY fruit stages included *GGPPS*, *PSY*, Z-*ISO*, *CRTISO* and *LCB*. Among the down-regulated pathway genes were *LCYE*, *BCH* and *ZEP* and showed that *PSY* mis-expression affected the expression of the other carotenoid pathway genes.

**FIGURE 4:**
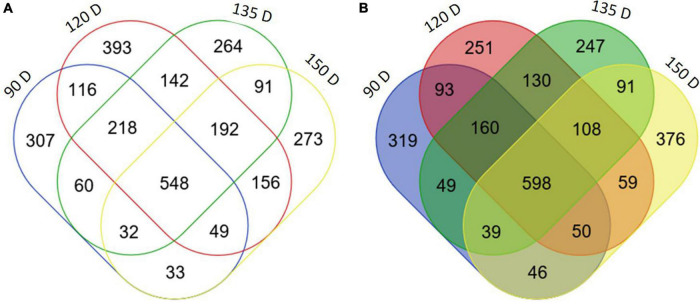
Differentially expressed genes in PSY transgenic apple fruit. Venn diagram of number of differentially expressed genes (log_2_ fold change ≥ 2, *P* < 0.05) between WT and OE-7 fruit at four developmental stages (90, 120, 135, 150 D). Upregulated **(A)**, downregulated **(B)** in PSY fruit flesh.

With a log_2_-fold cut-off ±2 (*p* < 0.05) and a false discovery rate threshold of *p* < 0.05, the number of upregulated and downregulated DEGs in PSY fruit compared with WT control were: 90 D (1354 up, 1363 down), 120 D (1449 up, 1814 down), 135 D (1422 up, 1547 down) and 150 D (1367 up, 1374 down) (Figures 4A,B). In comparison, the number of DEGs common to two or more fruit stages was 1637 upregulated and 1423 downregulated. However, since DEGs common to all four fruit stages are likely to include genes specifically responding to the perturbation caused by *PSY1* mis-expression, as well as any subsequent metabolic changes, the 548 upregulated and 598 downregulated genes common to all four fruit developmental stages, referred to as *PSY1*-associated genes (PSYAGs; Supplementary Table S3), were further examined. The most upregulated PSYAG was a BAG family molecular chaperone regulator (MD01G1073600), a homolog of AtBAG7, implicated in unfolded protein responses. Upregulated genes included 1-aminocyclopropane-1-carboxylate oxidase 1 (MD15G1205100), squalene monooxygenase, and transcription factors such as ethylene responsive factors (MdAP2D2, MD08G1060000), WRKY75 (MD09G1008800, similar to AtWRKY75, implicated in anthocyanin accumulation during stress), a homolog of AtMYB15, implicated in lignin biosynthesis, SEPALLATA1 (AGAMOUS-like 2) and a heat shock MYB close to BOS1. Among the downregulated PSYAGs were auxin-responsive proteins, beta-carotene isomerase D27, floral homeotic protein AGAMOUS and topless-related protein, transcriptional repressors, a senescence-related gene (SRG1) and MYB91, a homolog of Antirrhinum PHANTASTICA. To gain insight into the processes responding to the *PSY1* expression in apple fruit, we analyzed gene ontology (GO) term enrichment of the PSYAGs in the biological process (BP) and molecular function (MF) categories. The results showed enrichment for processes such as the carbohydrate metabolic process, lipid and fatty acid metabolic processes (BP category), and DNA binding transcription factor, catalytic and hydrolase activities in the MF category (Supplementary Figure S4).

### Weighted gene co-expression network analysis identified clusters of genes associated with carotenoid accumulation in apple

Weighted gene co-expression network analysis (WGCNA) identified clusters of genes that were highly correlated with three key traits: TCC, beta-carotene content, and fruit development stage. Using a *p* < 0.05 cut-off, three clusters (Brown, Green, and Red modules) showed significant relationships across all the three traits. The Brown and Green-coded modules displayed positive gene significance, which is based on the correlation of the gene expression profile with sample trait (Langfelder and Horvath, 2008), and the Red-coded module negative gene significance, with respect to total carotenoid accumulation (Supplementary Figure S5). The Brown-coded module had 1504 genes showing gene significance (GS) ≥ 0.6, and contained the carotenoid genes *DXS*, *PDS* and *Z-ISO*, as well as *BCH*, *ZEP* and *NCED5*. The Green-coded module had 1770 members with a GS ≥ 0.6 and included carotenoid-related genes such as *ZDS*, *BCH, LUT5* and *OR*. The presence of known carotenoid genes in these modules suggests the other genes in these sets are likely to be associated with carotenoid metabolism and thus provide the opportunity to discern transcriptional regulation of this metabolic pathway.

**FIGURE 5:**
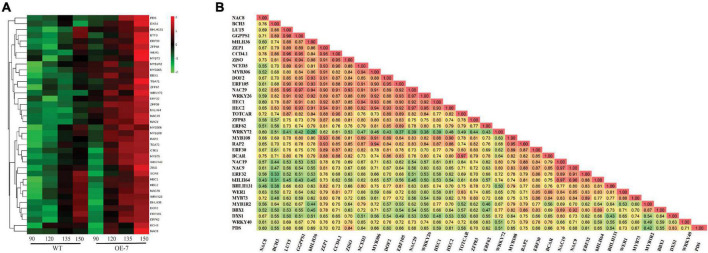
Analysis of carotenoid-associated transcription factor genes. **(A)** Heat map of differentially expressed transcription factor genes (Supplementary Table S4) in apple fruit flesh during development. The average gene expression of biological replicates at each of the four stages: 90, 120, 135, and 150 D, from WT and PSY transgenic line OE-7, were clustered using Euclidean distance relationships. Green to red color gradient indicates low to high relative gene expression. **(B)** Correlation matrix of gene transcripts and carotenoid metabolites (β-carotene, BCAR; total carotenoid content, TOTCAR) in transgenic PSY fruit. Values represent correlation coefficients with color gradient from green to red indicating weak to strong correlations at *P* < 0.05.

To identify TFs that were associated with apple carotenoid biosynthesis, we performed a cluster analysis of the carotenoid and TF genes in the Brown-coded module, using the average expression values of biological replicates and applying a GS cut-off > 0.6, which identified 34 TFs (Figure 5A and Supplementary Table S4). A Pearson correlation matrix between these genes and carotenoid contents further highlighted the relationship between these candidate TFs and carotenoid metabolism (Figure 5B). The apple *PDS* is a single-copy gene in the duplicated *M*. *domestica* genome and expected to have an important limiting role in the apple carotenoid pathway (Velasco et al., 2010; Daccord et al., 2017). *PDS* expression in fruit flesh was strongly correlated with *ZISO* (*r* = 0.84) and *DXS* (*r* = 0.83), suggesting these genes may be co-regulated Among the TF genes, *PDS* showed highest correlation with *NAC19* (*r* = 0.76) and high correlations with *ZISO* (*r* = 0.69) and *DXS* (*r* = 0.80), respectively, suggesting that *DXS*, *PDS* and *ZISO* may be co-regulated in apple fruit.

### *PSY1* expression increased fruit carotenoid content in the absence of light

**FIGURE 6:**
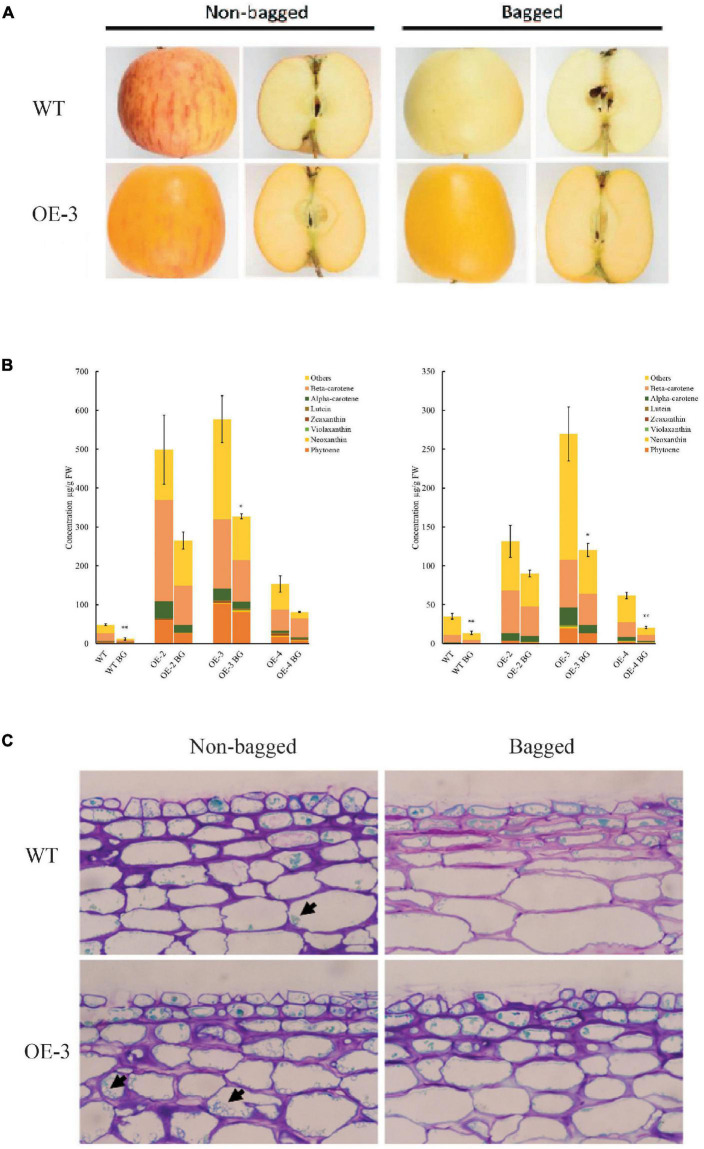
*PSY1* increased fruit carotenoid content in apple in the absence of light. **(A)** Representative bagged and non-bagged fruit of WT and PSY line OE-3 at 150 D. **(B)** Graphs showing carotenoid content in fruit skin (left) and flesh (right) of different PSY lines (OE-2, OE-3, OE-4) and WT. Error bars represent standard error of total carotenoid contents of three biological replicates. Bar graph with asterisk show significant difference from the non-bagged fruit of the same line using Dunnett’s test (*P < 0.05; **P < 0.01). **(C)** Stained fruit sections of WT and OE-3 displaying plastids (arrowed) in the cells.

To gain insight into how *PSY1* overexpression interacts with the effects of light on carotenoid accumulation in apple, we selected another three PSY lines, OE-2, OE-3, and OE-4 (which had sufficient numbers of fruit), and WT control, and bagged their fruit at 30 D until 150 D. Both bagged and non-bagged PSY fruit showed yellow skin color, with little or no red anthocyanin pigmentation. The flesh of PSY fruit, irrespective of bagging treatment, also showed yellow pigmentation, suggesting increased carotenoid accumulation (Figure 6A). In contrast, the bagged WT fruit had reduced pigmentation in the skin and flesh compared with the non-bagged WT fruit, which had skin with red stripes on a yellow background and a creamy flesh (Figure 6A). HPLC data showed there were significant changes to fruit carotenoid content and profile because of the bagging treatment (Figure 6B). In fruit skin, bagging reduced TCC ∼four-fold in the WT control (from 48 ± 2.3 to 12.1 ± 2.9 μg/g FW), and by ∼two-fold in the PSY lines OE-2 (498.8 ± 88.6 to 264.9 ± 21.7 μg/g FW), OE-3 (577.3 ± 60.7 to 327.2 ± 6.5 μg/g FW), and OE-4 (153.3 ± 21.2 to 81.5 ± 1.2 μg/g FW). Similarly, bagging reduced TCC in fruit flesh by 2.5-fold in the WT control (from 35.1 ± 3.6 to 13.9 ± 2.3 μg/g FW), while in PSY lines, TCC reduced by 1.5- to 3-fold in OE-2 (131.8 ± 20.8 to 90.2 ± 4.4 μg/g FW), OE-3 (269.7 ± 34.7 to 120.5 ± 8.5 μg/g FW), and OE-4 (62.0 ± 4.4 to 20.6 ± 1.4 μg/g FW). Overall, bagging reduced TCC in fruit skin and flesh of both WT and PSY lines. However, *PSY1* overexpression still significantly induced carotenoid accumulation in these tissues even in the absence of light. The reduced carotenoid content in bagged fruit was consistent with the reduced numbers of plastids in these tissues. Bright-field microscopy analysis showed fewer plastids in bagged PSY and WT fruit compared with their respective non-bagged fruit, while in both bagged and non-bagged fruit, PSY tissues showed more plastids than the WT (Figure 6C).

### Transcriptional changes in bagged fruit flesh

To ascertain transcriptional changes associated with the bagging treatment, the transcriptome of 150 D fruit flesh from three PSY lines OE-2, OE-3, OE-4, and the WT was analyzed. With a log_2_-fold cut-off ± 2 (*p* < 0.05) and a false discovery rate threshold of *p* < 0.05, bagged versus non-bagged WT fruit had 335 DEGs (152 up, 182 down), compared with 136 DEGs (34 up, 132 down) in bagged PSY fruit, present in at least two OE lines. Twenty-three of these DEGs (11 up, 12 down) were present in both bagged WT and PSY fruit. In contrast, when comparing WT and PSY fruit (bagged and non-bagged) we identified 530 upregulated and 1500 downregulated genes in PSY lines, a snapshot of a PSY-induced transcriptional response. Bagging also reduced expression of known light-regulated genes such as *ELONGATED HYPOCOTYL 5* (*HY5)*, *chlorophyll a-b binding protein*, phytochrome a and *ribulose-l,5-bisphosphate carboxylase oxygenase*.

**FIGURE 7:**
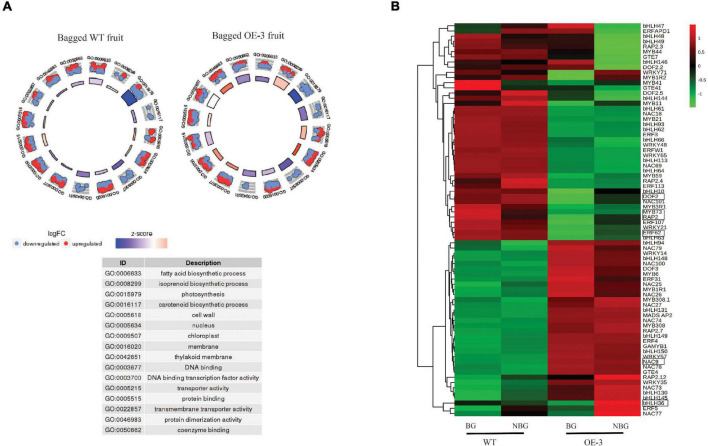
Differentially expressed genes (DEGs) in bagged apple fruit. **(A)** Go Plot R of DEGs in bagged fruit of WT and OE-3 fruit. The scatter plots show log_2_ fold change (logFC) for each gene under the gene ontology (GO) number. Red dots indicate upregulated genes and blue dots show downregulated genes. The *z*-score bars are a measure of the extent to which each identified process is upregulated or downregulated. **(B)** Heat map of differentially expressed transcription factor genes in bagged fruit. Clustering done using the average expression values of three biological replicates of bagged (BG) and non-bagged (NBG) fruit of WT and OE-3 transgenic line. Green to red color gradients indicate low to high relative gene expression. The highlighted TFs are common to the bagging and fruit development (Figure 5A) data sets.

Assessment of the 54 selected carotenoid and related genes (Supplementary Table S2) in WT fruit revealed the expression of *DXS*, *GGPPS*, *ZDS*, *CRTISO*, *BCH*, *ZEP*, *VDE*, and *ECH* was downregulated in the bagged WT fruit. This downregulation of carotenoid genes was reversed in the PSY fruit. To ascertain whether *PSY1* modulated the expression of these genes in the absence of light, we compared bagged WT with bagged PSY fruit of the three independent OE lines. The expression of *DXS*, *GGPPS*, *PDS*, *ZDS*, *CRTISO*, *BCH*, and *ZEP* was upregulated in bagged PSY fruit of all or at least two transgenic lines, again revealing a feedforward effect on the pathway genes by *PSY1* mis-expression, independent of light. GO analysis of both WT and OE-3 DEGs showed enrichment for fatty acid biosynthesis (GO:0006633), photosynthesis (GO:0015979) and carotenoid biosynthesis (GO:0016117), all of these biological processes had negative z scores indicating their downregulation due to bagging treatment (Figure 7A).

To identify TFs associated with the *PSY1*-induced gene expression response in bagged fruit, differentially expressed TF genes (Supplementary Table S5) were selected for cluster analysis, using their average expression values. These divided into two clusters and clearly differentiated between WT and PSY fruit (Figure 7B). These TFs compared with those differentially expressed during fruit development of WT and PSY lines (Figure 5A) identified six TFs common to both data sets: *MdbHLH36* (MD09G1233000), *MdDOF2* (MD05G1018200), *MdRAP2* (MD17G1152400), *MdERF62* (MD04G1009000), and *MdMYB73* (MD15G1288600) and *MdNAC9* (MD01G1093500).

**FIGURE 8:**
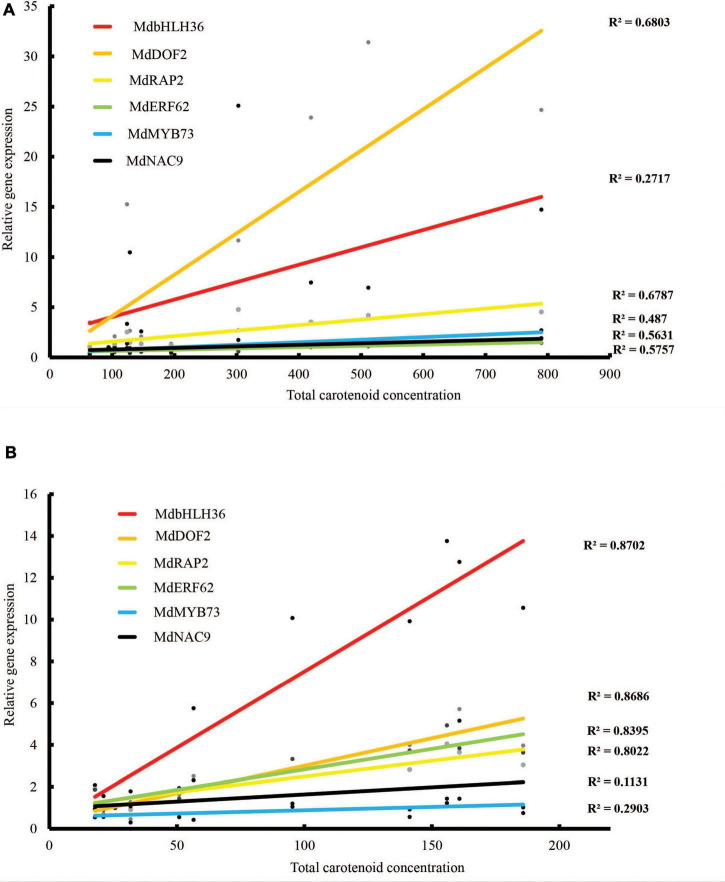
Correlations between carotenoid content and TF gene expression as determined by qRT-PCR in **(A)** fruit skin and **(B)** fruit flesh of WT and PSY fruit. *R*^2^ values indicate coefficient of determination.

Expression of these TFs was validated using qPCR of fruit skin and flesh from different PSY transgenic lines. The expression of these genes, in both fruit skin and flesh, showed an increasing trend during fruit development that was consistent with the carotenoid accumulation in these tissues. Their expressions were also higher in the PSY lines compared with the WT at almost all the fruit stages examined (Supplementary Figure S6). Pearson correlation analysis showed strong correlation between the expression of these genes and carotenoid accumulation in both fruit skin and flesh tissues (Figures 8A,B). The gene expression of *MdbHLH36* was highly correlated with total carotenoid accumulation in the fruit flesh (*r* = 0.93), but had reduced correlation in the skin (*r* = 0.52). In contrast, *MdDOF2* and *MdRAP2* were highly correlated with carotenoid accumulation in both fruit skin (*r* = 0.82, 0.82) and flesh (*r* = 0.93, 0.90), respectively (Supplementary Table S6). Overall, the identification and expression of these TF genes suggest that they may have important roles during carotenogenesis in apple.

## Discussion

### Phytoene synthase controls carotenoid biosynthesis in apple

Phytoene synthase controls an important step, and acts as a limiting factor, in the carotenoid pathway (Rosas-Saavedra and Stange, 2016; Ahrazem et al., 2019). In this study, we analyzed *PSY1* overexpression in apple fruit to understand carotenoid regulation in this crop species. A deep yellow fruit pigmentation phenotype was attributed to increased carotenoid compounds in both skin and flesh tissues, with beta-carotene as the predominant compound. The accumulation of these compounds was probably due to an increased pathway flux, since the precursor GGPP is a common substrate for the chlorophyll, gibberellin and tocopherol pathways (Okada et al., 2000; Zhou et al., 2017). This altered pathway flux was illustrated with regenerated PSY leaf calli, which in the presence of the PDS inhibitor norflurazon, showed increased phytoene accumulation (Schaub et al., 2018). The increased phytoene content in PSY calli, with and without NFZ treatment, clearly indicated that *PSY1* overexpression in apple increased pathway flux in these tissues. Interestingly, apple has six *PSY* genes (compared with a single copy in Arabidopsis), with multiple *PSYs* expressed in fruit tissues. The increased carotenoid accumulation in response to *PSY1* overexpression confirms that the first committed enzyme step is a limiting factor in apple (Ampomah-Dwamena et al., 2015).

The enhanced carotenoid content in PSY fruit was associated with accelerated plastid transition and increased abundance of chromoplasts, as revealed by confocal microscopy. This metabolite-induced chromoplast abundance is probably mediated by OR, which was found in our weighted gene correlation network analysis (WGCNA), and has a role in plastid-induced carotenoid accumulation (Lu et al., 2006; Chayut et al., 2017; Feder et al., 2019). The effect of increased storage capacity on carotenoid accumulation is well established (Osorio, 2019). Plastid differentiation into chromoplasts stimulates carotenoid accumulation, as first revealed in the cauliflower orange mutant, where a gain-of-function mutation led to over-accumulation of beta-carotene (Lu et al., 2006). Overexpression of the *OR* gene has subsequently become an alternative approach to pathway engineering for increasing carotenoid content in crop plants like potato, rice, maize and tomato (Lopez et al., 2008; Bai et al., 2014; Park et al., 2016; Berman et al., 2017). The post-transcriptional regulation of PSY protein levels by OR and OR’s effect on downregulation of beta-carotene hydroxylase further highlights the crosstalk between the various mechanisms controlling carotenogenesis. Our results show PSY-induced carotenoid biosynthesis was accompanied by accelerated chromoplast differentiation and increased expression of apple *OR* genes in the PSY fruit. This further reinforces the connection between plastid differentiation and carotenoid biosynthesis pathways in plants (Shumskaya et al., 2012; Park et al., 2016; Welsch et al., 2018).

### Phytoene synthase overexpression increased carotenoid content in bagged fruit

Bagging of apple fruit reduced fruit carotenoid content and plastid concentration, highlighting the light regulating component of carotenoid accumulation and plastid development. Light plays a significant role in regulating fruit pigmentation, acting through signaling factors to regulate gene expression (Llorente et al., 2017). The light exclusion effect on the apple fruit tissues was associated with reduced gene expression of *HY5* and Phytochrome A (*PHYA*), which are two key light signaling regulators whose functions have been studied in tomato. The loss of function of SlHY5, either through genome editing or RNAi downregulation of HY5, impaired fruit ripening and resulted in reduced carotenoid content (Liu et al., 2004; Wang W. et al., 2021). Phytochromes, such as PHYA and PHYB, mediate light-induced ripening and carotenoid accumulation in tomato fruit with their expression level positively correlated with carotenoid biosynthesis, though such effect could be fruit stage specific or may involve complex interactions with other phytochromes (Alba et al., 2000; Gupta et al., 2014; Bianchetti et al., 2018). While light exclusion negatively affected fruit carotenoid content, the bagged PSY apple fruit still accumulated higher carotenoid content than the bagged WT suggesting *PSY1* expression could compensate for the negative effect of light exclusion on the carotenoid pathway. This is consistent with PSY as a key target for light regulation of carotenoid biosynthesis, acting through transcription factors such as PIF1, which negatively regulates *PSY* (Toledo-Ortiz et al., 2010). Under low light conditions, PIF1 expression in tomato increases and binds the *PSY* promoter to repress gene expression (Llorente et al., 2016), implying increased *PSY* expression could override the inhibition of carotenogenesis by light exclusion.

### Transcriptional response to *PSY1* expression in fruit

The transcriptional changes induced by *PSY1* overexpression provided an opportunity to identify related genes associated with the phenotypic changes. The observed up- and down-regulation of carotenoid pathway genes was consistent with the metabolite accumulation pattern, with beta-carotene as the dominant compound in PSY fruit. Genes encoding PDS, ZISO, ZDS, CRTISO and LCB showed increased gene expression in PSY fruit compared with the WT, and conversely the expression of *BCH*, *ECH*, and *ZEP* was reduced. The accumulation of beta-carotene, and not phytoene, in the transgenic fruit tissues clearly indicated an active apple carotenoid pathway, with increased gene expression resulting in increased enzyme activity. *PDS* and *ZISO*, for example, are both single-copy genes in the apple genome and their increased activities would be required for the subsequent conversion of phytoene to zeta carotene (Chen et al., 2010; Beltrán et al., 2015; Rodrigo et al., 2019).

The changes in carotenoid gene expression suggested PSY1-induced feedforward regulation of the apple carotenoid pathway. The plant carotenoid pathway is characterized by metabolic feedforward and feedback regulation (McQuinn et al., 2015). *PSY* overexpression in Arabidopsis elevated *DXS* transcripts, which suggested a positive feedback regulation of the MEP pathway (Rodríguez-Villalón et al., 2009). In contrast, the over-accumulation of phytoene in the *pds3* Arabidopsis mutant caused negative feedback regulation of the carotenoid pathway, decreasing *GGPPS*, *PSY*, *ZDS*, and *LCYB* expression in the mutant (Qin et al., 2007). The expression of bacterial *CrtI* gene in tomato mutants elevated the pool of lycopene substrate in fruit and caused a feedforward transcriptional activation of the downstream cyclase genes (Enfissi et al., 2017). Similarly, overexpression of Arabidopsis *PDS* triggered a feedforward effect on carotenoid gene expression in tomato leaves and flowers (McQuinn et al., 2018). *LCY-B* overexpression increased *PSY* transcripts in transgenic carrot (Moreno et al., 2013); conversely, overexpression of carotene hydroxylase CYP97A3 reduced PSY protein content in carrot, suggesting a negative feedback regulation (Arango et al., 2014). Although mechanisms involved in these carotenoid feedback regulations are mainly uncharacterized, apocarotenoid-derived signaling molecules are involved in transcriptional regulation of carotenoid genes and inhibition of enzymatic activity (Avendaño-Vázquez et al., 2014; Enfissi et al., 2017). Whilst gene transcript levels are not always indicative of enzyme activity, it is possible *PSY1*-induced accumulation of phytoene exerted a metabolic feedforward regulation of carotenoid pathway genes that subsequently led to increased accumulation of downstream compounds, such as beta-carotene.

### *PSY1* overexpression uncovers carotenoid-associated regulatory genes

The transcriptional response to *PSY1* overexpression is likely to be mediated by transcription factors, whose gene expression would be expected to correlate with their target genes, as identified using gene co-expression analysis (Langfelder and Horvath, 2008; Zhang et al., 2020; Zogopoulos et al., 2021). Our analysis found six carotenoid-associated TF genes with their expression strongly correlated with fruit carotenoid accumulation during on-tree development as well as in the absence of light. These TF genes belong to the bHLH, DOF, ERF, MYB, and NAC families, some of which have members demonstrated to regulate carotenoid metabolism (Stanley and Yuan, 2019).

We showed previously that kiwifruit *AdMYB7* plays a significant role in controlling chlorophyll and carotenoid accumulation during fruit development through regulation of certain key genes (Ampomah-Dwamena et al., 2019). Other MYBs from *Erythranthe lewisii* (ElRCP1), citrus (CrMYB68), and Medicago (WP1) have been implicated in carotenoid regulation, although they are phylogenetically distant (Sagawa et al., 2016; Zhu F. et al., 2017; Meng et al., 2019). These examples suggest that MYBs from different clades have evolved to control carotenoid biosynthesis. *MdMYB73*, from this study, is an R2R3 MYB with similarity to Arabidopsis MYB70, MYB73, MYB77, and MYB44, which generally mediate responses to biotic and abiotic stresses (Stracke et al., 2001; Dubos et al., 2010). Apple *MdMYB73* controls malate metabolism through activation of pathway genes (Hu et al., 2017), while its Arabidopsis homolog, *AtMYB73*, regulates salt stress response (Kim et al., 2013; Wang L. et al., 2021). In addition to MYBs, bHLH TFs have a role in controlling carotenoid metabolism. BHLHs, such as the Phytochrome interacting factor 1 (PIF1), are key mediators of carotenogenesis in Arabidopsis, directly binding the promoter of *PSY* to repress gene expression under low light conditions, albeit this role was not required in roots (Toledo-Ortiz et al., 2010; Ruiz-Sola et al., 2014). In tomato, *SlPRE2*, a gibberellic acid-induced bHLH TF, negatively regulates carotenogenesis by downregulating *PSY* and *ZDS* gene expression (Zhu Z. et al., 2017; Zhu et al., 2019). Other bHLHs positively regulate carotenoid accumulation, such as papaya CpbHLH2, which activated expression of *CYCB* and *LCYB* in transient assays, and SlARANCIO, a tomato bHLH gene associated with a carotenoid QTL (D’Amelia et al., 2019; Zhou et al., 2019). Fine mapping of the Y2 locus controlling beta-carotene accumulation in carrot found a differentially expressed bHLH TF, suggesting a potential regulatory role in carotenoid metabolism (Ellison et al., 2017).

NAC domain (derived from the three type members, NO APICAL MERISTEM, ATAF, and CUP-SHAPED COTYLEDON; Aida et al., 1997) TFs regulate diverse traits such as stress responses, senescence, fruit ripening events and carotenoid biosynthesis in fruit. The tomato NAC ripening regulator NOR-like 1 was shown to directly regulate genes involved in ethylene biosynthesis as well as the carotenoid-related genes *GGPPS2* and *SGR1* (Gao et al., 2018). GGPPS is responsible for the synthesis of GGPP, which is the substrate for PSY, while SGR1 negatively regulates PSY to control carotenoid accumulation in tomato (Luo et al., 2013). The modulation of SlNAC1 transcript levels led to changes in carotenoid accumulation patterns, suggesting it was a key regulator. Overexpression of this gene resulted in delayed fruit ripening and reduced carotenoid content (Ma et al., 2014). Conversely, downregulation of SlNAC1 produced higher total carotenoid and lycopene in fruit, although fruit ripening was also delayed (Meng et al., 2016). This is in contrast to *SlNAC4*, which positively regulated carotenoid accumulation; RNAi repression resulted in downregulation of *SlPSY1* transcripts and reduced carotenoid content (Zhu et al., 2014). In papaya, CpNAC1 activated the promoters of phytoene desaturase genes both *in vitro* and in transient assays (Fu et al., 2016). The AP2/ERFs also have roles in plant carotenoid metabolism. One of the two AP2/ERFs identified in this study, MdRAP2 (AP2D11), previously activated the *MdPSY2* promoter in a transient promoter assay screen suggesting that it is a positive regulator of the carotenoid pathway (Ampomah-Dwamena et al., 2015). Its Arabidopsis homolog, AtRAP2.2 interacted with *PSY* and *PDS* upstream regulatory sequences *in vitro*, indicating it may be part of a regulatory regime controlling carotenogenesis (Welsch et al., 2007). In a more recent study, overexpression of another AP2/ERF, *MdAP2*-*34*, in apple increased fruit carotenoid content, with the TF able to bind and activate the *MdPSY2* promoter (Dang et al., 2021). In tomato, SlAP2a and SlERF6 play important roles in carotenogenesis acting as negative regulators (Chung et al., 2010; Karlova et al., 2011; Lee et al., 2012). While the gene expression of the apple TFs in this study strongly correlated with fruit carotenoid accumulation, further experimentation will be required to characterize their functional roles in carotenogenesis.

In summary, overexpression of *PSY1* in apple produced fruit with increased carotenoid content, accelerated plastid differentiation, a feedforward effect in the pathway and a transcriptomic response that included altered expression of key TFs whose expression suggested roles in carotenogenesis and utility for developing biofortified apple cultivars.

## Materials and methods

### Cloning and plant materials

The apple *phytoene synthase 1* (MD17G1133400) was amplified from fruit complementary DNA (cDNA) using Hi-fidelity Taq polymerase (Life Technologies, Carlsbad, CA, United States) and primers PSY1-F and PSY1-R (Supplementary Table S1). The PCR amplicon was initially cloned into PCR8/GW TOPO vector (Life Technologies, Carlsbad, CA, United States) for sequence confirmation and then cloned into the binary vector pHEX2 using the Gateway cloning strategy as previously described (Ampomah-Dwamena et al., 2019). *Agrobacterium* strain LBA4404 carrying the resulting vector, pHEX2S-MdPSY1, was used to transform ‘Royal Gala’ apple as described previously (Yao et al., 1995). Transgenic ‘Royal Gala’ shoots from tissue culture were grafted onto ‘Malling 9’ apple rootstock for growth in soil in glasshouse. Flowers at anthesis were hand pollinated with ‘Granny Smith’ pollen. Fruit harvested at different development stages [90, 120, 135, and 150 days after pollination (D)] and separated into skin and flesh (cortex) tissues were frozen in liquid nitrogen for pigment analysis and gene expression. For the light exclusion experiment, fruit at 20 D were covered in paper bags, and left to grow on the tree until 150 D when they were harvested and analyzed. For callus culture, young leaves were collected from three PSY lines (OE-2, OE-3, OE-4), sterilized and cultured on callus induction medium (MS basal medium + 1 mg/L BA + 0.5 mg/L NAA, 7% agar), supplemented with 0.3 mg/L norflurazon, for 2 weeks.

### High-performance liquid chromatography pigment analysis

Carotenoid and chlorophyll pigments were extracted from fruit tissues using acetone as described previously (Ampomah-Dwamena et al., 2015). Samples were weighed into tubes and solvent extracted overnight. HPLC analysis was performed using a Dionex Ultimate 3000 solvent delivery system (Thermo Scientific, Waltham, MA, United States) with a photodiode array detector was fitted with Acclaim C30 column (5 μm, 250 × 4.6 mm), coupled to a C30 guard column (Thermo Scientific, Waltham, MA, United States) as previously reported (Ampomah-Dwamena et al., 2019). Phytoene was monitored at 280 nm, colored carotenoids and Chlb were detected at 450 nm, while Chla and its derivatives were monitored at 400 nm. Carotenoid contents were expressed as b-carotene equivalents per gram dry weight (DW) of tissue. All *trans*-β-carotene, lutein and Chla standards were purchased from Sigma Chemicals (Sigma-Aldrich, St Louis, MO, United States). Other carotenoids were putatively identified by comparison with reported retention times and spectral data.

### RNA isolation and complementary DNA synthesis

Total RNA was extracted from tissues using the Spectrum RNA isolation kit (Sigma-Aldrich, St Louis, MO, United States) using a modified manufacturer’s protocol. One gram of homogenized tissue in liquid nitrogen was used as the starting material and the volumes of buffers were increased accordingly. cDNA was synthesized from total RNA using the Quantitec reverse transcription kit (Qiagen, Hilden, Germany) following the manufacturer’s protocol. RNA samples were treated with the genomic DNA wipeout buffer followed by reverse transcription reaction as described earlier (Ampomah-Dwamena et al., 2019). The reactions were incubated at 42°C for 30 min followed by 95°C for 3 min for enzyme inactivation.

### Quantitative real-time PCR analysis

First-strand cDNA samples from fruit skin and flesh tissues at 90, 120, 135, and 150 D were diluted 1:20 and used as templates for quantitative real-time PCR according to methods described previously (Ampomah-Dwamena et al., 2015, Ampomah-Dwamena et al., 2019). PCR analysis was performed using the LightCycler 1.5 system and the SYBR Green master mix (Roche, Mannheim, Germany), following the manufacturer’s protocol. Each reaction sample was analyzed from biological replicates, with a negative control using water as template. PCR conditions were as follows: pre-incubation at 95°C for 5 min followed by 40 cycles each consisting of 10 s at 95°C, 10 s at 60°C and 20 s at 72°C. Amplification was followed by a melting curve analysis with continuous fluorescence measurement during the 65–95°C melt (Ampomah-Dwamena et al., 2019). The relative expression was calculated using LIGHTCYCLER software v.4 and the expression of each gene was normalized to reference genes *MdACTIN* and *MdEF1A*, which have been shown to have stable expression in these tissues (Nieuwenhuizen et al., 2013; Ampomah-Dwamena et al., 2015, Ampomah-Dwamena et al., 2019). Primers were designed using PRIMER3 software (Rozen and Skaletsky, 2000) with respective EST sequences as templates. Primers were subjected to a stringent set of criteria, with a minimum melting temperature of 60°C and at least 22 nucleotide length.

### Transcriptome analysis by RNA sequencing

Total RNA from fruit tissues with A260/280 and A260/230 absorbance ratios both greater than 1.8 were used in sequencing library preparation using the TruSeq mRNA library preparation kit (Illumina, NovogeneAIT Genomics, Singapore). Transcriptome was sequenced on a HiSeq2000 (Illumina) using 2 × 100 bp paired-end sequencing. Each treatment had three biological replicates, with each replicate being a pool of tissues from 5–7 fruit from individual plants. The multiplexed libraries were run on multiple lanes, generating between 40 and 56 million reads per sample. The resulting reads were then quality and adapter trimmed using bbduck from the BBMap suite (version 37.53), with a quality threshold cut-off of 20 and minimum nucleotide length of 35 bases, where k-mer size of 25 was used. Thereafter, reads were aligned to the ‘Golden Delicious’ double haploid annotated gene models (Daccord et al., 2017) using STAR (version 2.5.2b) and the ‘–quantMode GeneCounts’ flag was used to extract read counts for the annotated genes. Differentially expressed genes identified using DESeq2 analysis, with a cut-off probability of *p* < 0.05, were subjected to Gene ontology (GO) enrichment analysis using goseq package (Young et al., 2012) in R and thereafter the Go Plot R package (Walter et al., 2015) for visualization. Weighted gene correlation network analysis was performed using R package (Langfelder and Horvath, 2008) to identify modules highly associated with selected traits, and the networks visualized using Cytoscape 3.8.2 (Saito et al., 2012).

### Statistical analysis

Hierarchical clustering of gene expression and metabolite content data represented by the biological replicates was performed using MetaboAnalyst v 5.0^1^ (Pang et al., 2021). Gene expression data were organized with gene IDs in the rows and biological replicates in the columns. Data were normalized by Log transformation where required. Heat maps were generated using Euclidean distance of the average values of biological replicates. Correlation tables with *p*-value cut-off < 0.05 were generated in MetaboAnalyst as above and color was formatted in Microsoft^®^ Excel^®^.

### Microscopy

Sections of 120, 135, and 150 D apple fruit tissue [150–250 nm cut using a VT1000S vibratome (Leica Microscopy Systems Ltd., Heerbrugg, Switzerland)] were taken from the equatorial region of each apple, mounted in 0.1 M phosphate buffer and imaged using an FV3000 laser scanning confocal (Olympus Optical Co. Ltd., Tokyo, Japan) on an IX83 inverted microscope platform (Olympus Optical Co. Ltd., Tokyo, Japan). To quantify differences in carotenoid and chlorophyll intensity between different fruit, lambda scans were performed using a 488 nm laser for excitation, collecting stepwise auto fluorescence emissions between 500 and 750 nm with a bandwidth of 10 nm and a step size 5 nm. To visualize carotenoid and chlorophyll accumulation in fruit sections, a 30-μm z-stack at 1-μm steps was collected for spectral bands between 500 and 550 nm, and 650–700 nm, respectively. Images were processed using Olympus Fluoview software to give a maximum intensity projection (Olympus Optical Co. Ltd., Tokyo, Japan).

## Data availability statement

The datasets presented in this study and accession numbers are included in the article/Supplementary material. Further enquiries may be directed to the corresponding author.

## Author contributions

CA-D designed the research and wrote the draft manuscript. ST transformed the apple plants. AT analyzed the transcriptome data and network analysis. CE and NB carried out total RNA isolation, cDNA synthesis, and qPCR analysis. RR and PS carried out the light and confocal microscopy analysis. HI constructed mRNA libraries for transcriptome sequencing. AA and RE assisted with data analysis and provided helpful comments on manuscript. All authors contributed to the article and approved the submitted version.

## Footnotes

1
https://www.metaboanalyst.ca/MetaboAnalyst/home.xhtml

## Supplementary material

Supplementary Figure S1 Graph of carotenoid pigments accumulating in regenerated calli from wild type (WT) and PSY transgenic (OE) leaf tissues. Bars represent the average of three biological replicates ± SE.

Supplementary Figure S2 Gene expression of carotenoid genes in fruit skin of WT, OE-5, and OE-7 lines as determined by real-time qPCR during fruit development. Bars represent the average of three biological replicates ± SE. LCE, lycopene epsilon-cyclase; ECH, epsilon carotene hydroxylase; ZEP, zeaxanthin epoxidase.

Supplementary Figure S3 PCA plot of RNA-sequencing data (RPKM) of WT and OE-7 apple fruit flesh sampled at 90 (S1), 120 (S2), 135 (S3), and 150 (S4) D.

Supplementary Figure S4 Gene ontology (GO) term enrichment of the PSYAGs in the biological process (BP) and molecular function (MF) categories. Enriched categories are highlighted in yellow.

Supplementary Figure S5 **(A)** Heatmap and eigengene expression of brown (left) and green (right) modules, across apple fruit development. **(B)** Module membership-gene significance correlation (left) and multi-dimensional plots (right) of brown (top panel) and green (bottom panel) modules.

Supplementary Figure S6 Gene expression analysis of the six carotenoid-associated TFs. Relative expression as determined by RT-PCR in fruit skin and flesh of WT, OE-5, and OE-7 lines during fruit development. Bars represent the average of three biological replicates ± SE.

Supplementary Table S1 List of PCR primers used.

Supplementary Table S2 List of 54 apple carotenoid genes identified from the “GDDH” apple sequence database (Daccord et al., 2017).

Supplementary Table S3 List of PSY-associated apple genes.

Supplementary Table S4 List of differentially expressed TFs (GS > 0.6) during apple fruit development.

Supplementary Table S5 List of differentially expressed TFs in bagged apple fruit. Highlighted in bold are the six TF genes common to both bagging and fruit development datasets.

Supplementary Table S6 Correlation coefficient (*r*) of the relationship between gene expression of carotenoid-associated TFs, apple carotenoid genes and carotenoid content in fruit skin and flesh. Color gradient from red to green, with arrows, indicate weak to strong correlations.
