# Supplementary figures and images for: Overexpression of PSY1 increases fruit skin and flesh carotenoid content and reveals associated transcription factors in apple (Malus × domestica)
Source: Front Plant Sci. 2022 Sep 15;13:967143. doi: 10.3389/fpls.2022.967143 (PMC9520574; doi:10.3389/fpls.2022.967143)

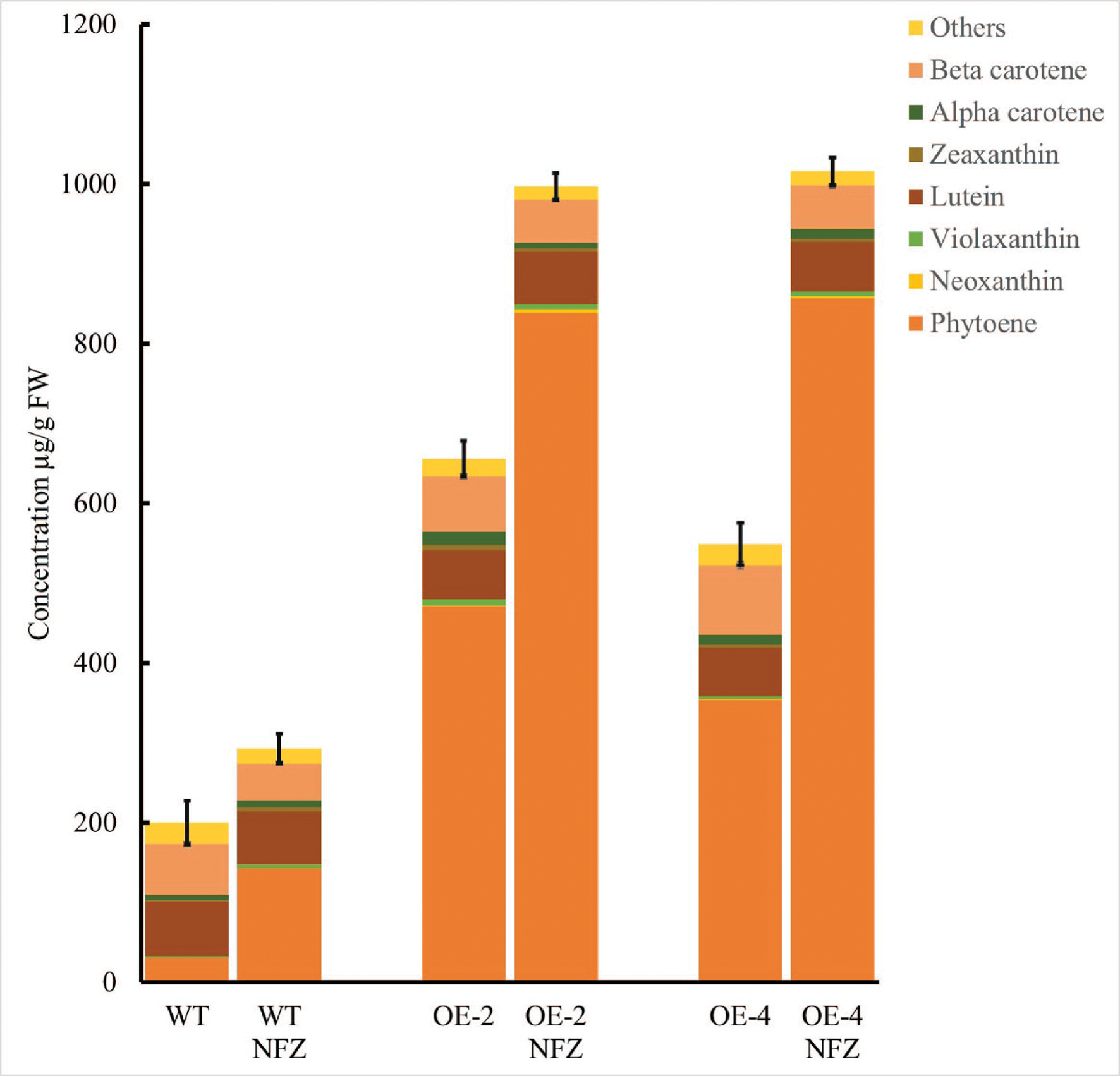

Supplement: Supplementary Figure S1 — Graph of carotenoid pigments accumulating in regenerated calli from wild type (WT) and PSY transgenic (OE) leaf tissues. Bars represent the average of three biological replicates ± SE. [file Image_1.JPEG]

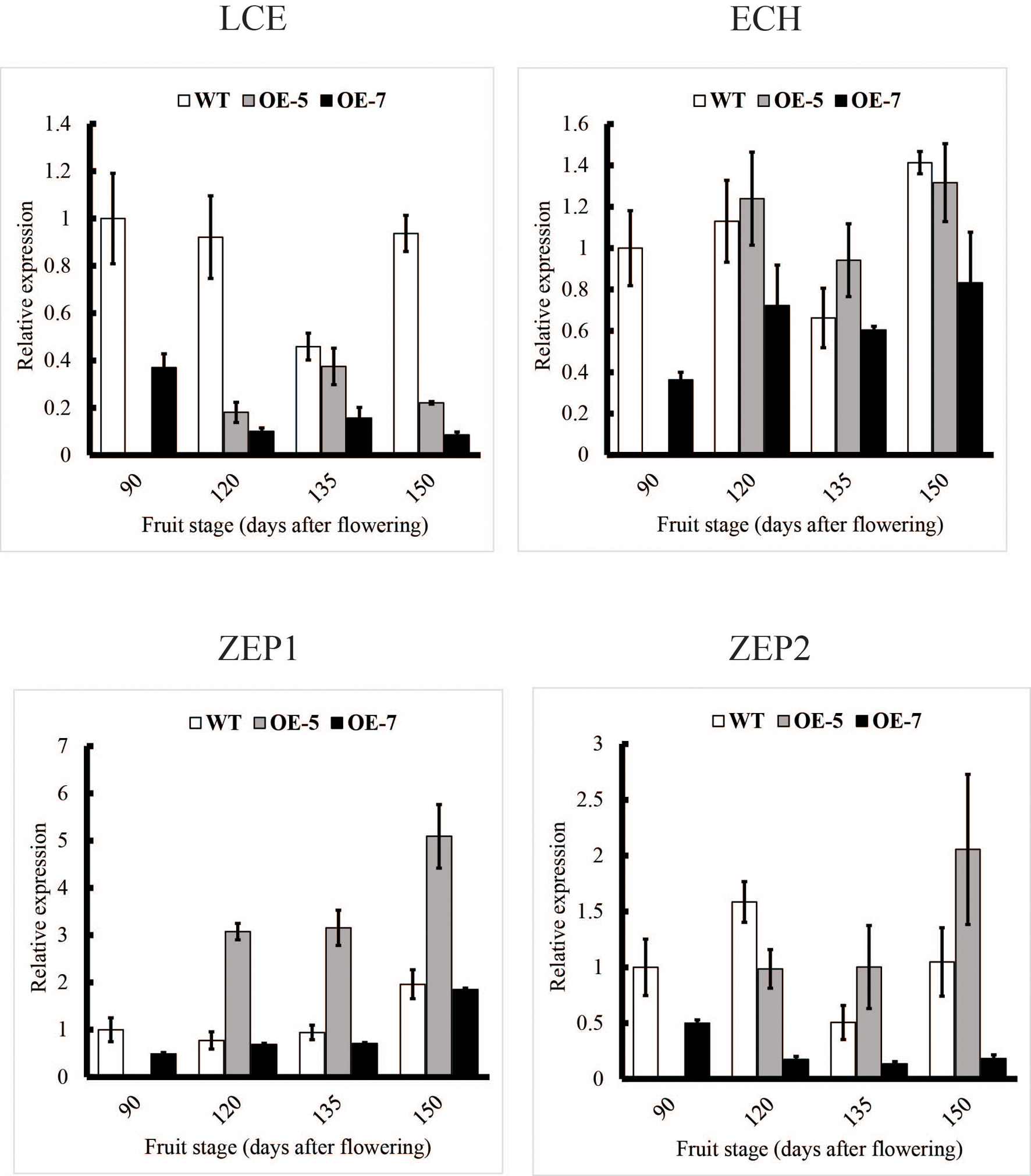

Supplement: Supplementary Figure S2 — Gene expression of carotenoid genes in fruit skin of WT, OE-5, and OE-7 lines as determined by real-time qPCR during fruit development. Bars represent the average of three biological replicates ± SE. LCE, lycopene epsilon-cyclase; ECH, epsilon carotene hydroxylase; ZEP, zeaxanthin epoxidase. [file Image_2.JPEG]

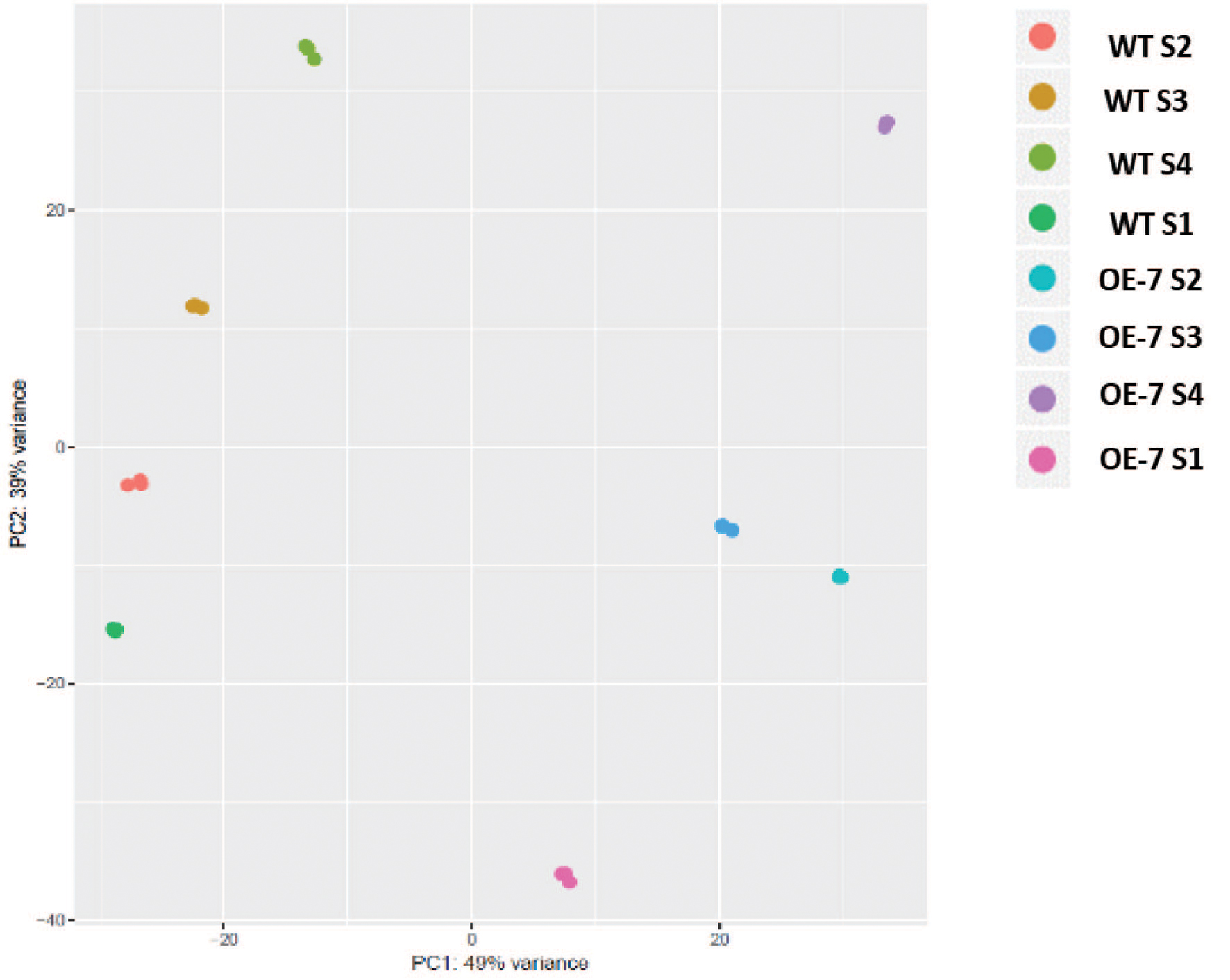

Supplement: Supplementary Figure S3 — PCA plot of RNA-sequencing data (RPKM) of WT and OE-7 apple fruit flesh sampled at 90 (S1), 120 (S2), 135 (S3), and 150 (S4) D. [file Image_3.JPEG]

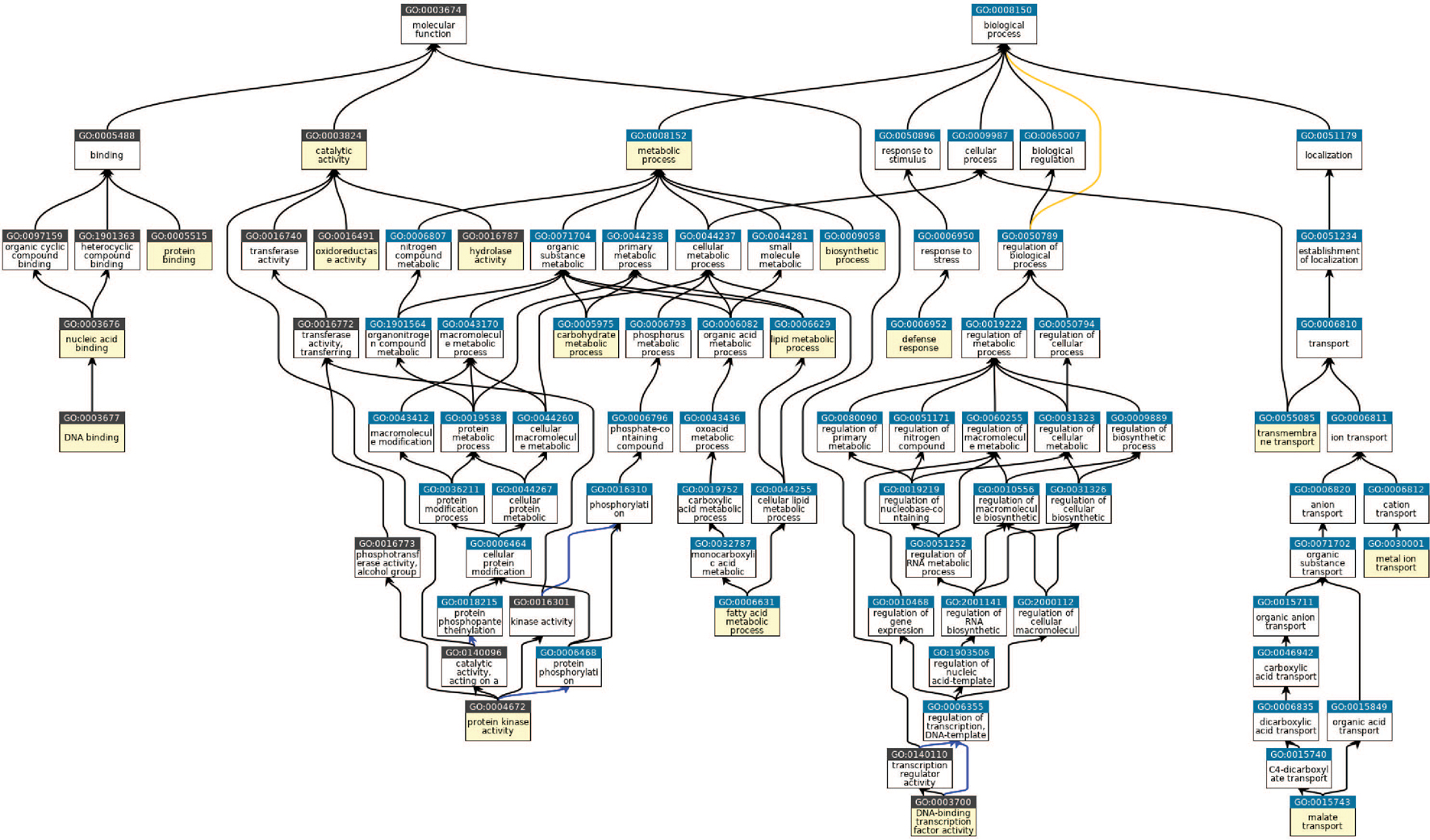

Supplement: Supplementary Figure S4 — Gene ontology (GO) term enrichment of the PSYAGs in the biological process (BP) and molecular function (MF) categories. Enriched categories are highlighted in yellow. [file Image_4.JPEG]

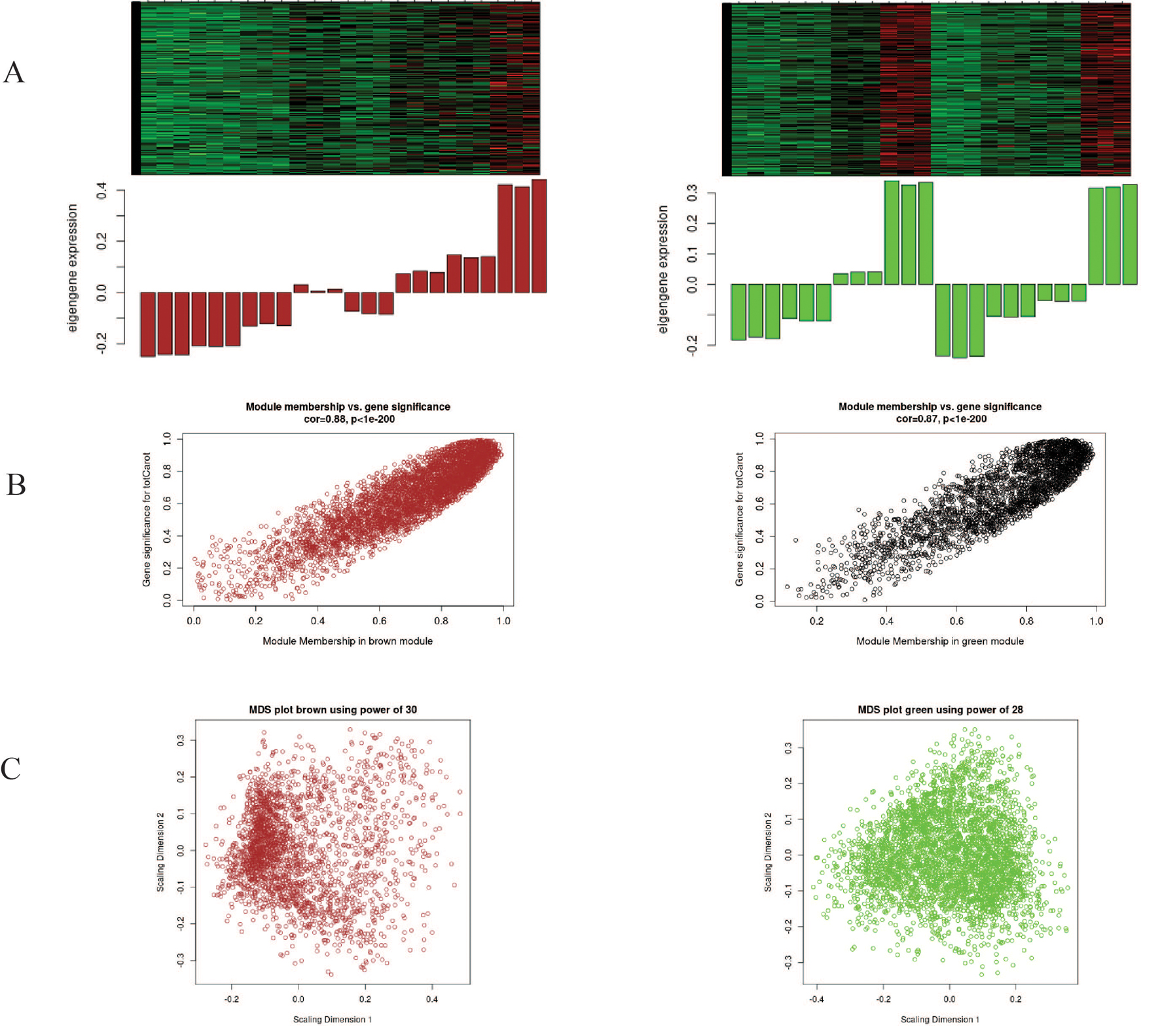

Supplement: Supplementary Figure S5 — (A) Heatmap and eigengene expression of brown (left) and green (right) modules, across apple fruit development. (B) Module membership-gene significance correlation (left) and multi-dimensional plots (right) of brown (top panel) and green (bottom panel) modules. [file Image_5.JPEG]

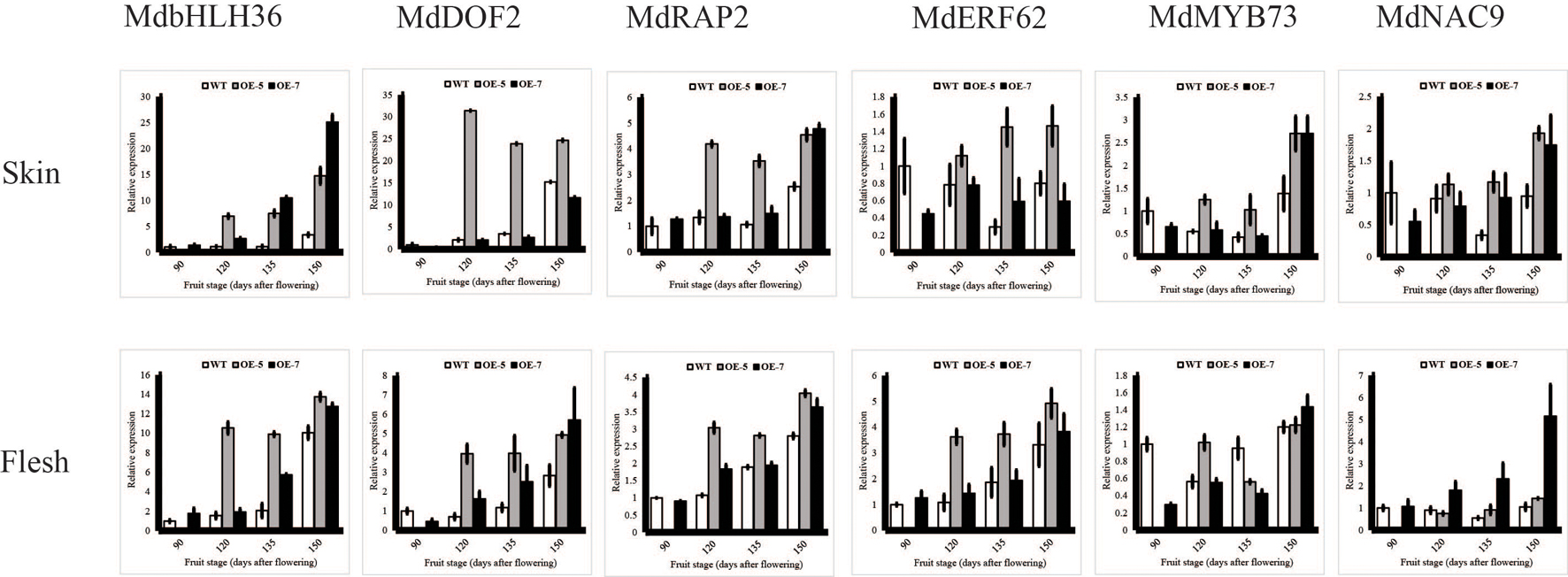

Supplement: Supplementary Figure S6 — Gene expression analysis of the six carotenoid-associated TFs. Relative expression as determined by RT-PCR in fruit skin and flesh of WT, OE-5, and OE-7 lines during fruit development. Bars represent the average of three biological replicates ± SE. [file Image_6.JPEG]
