# Supplementary material for: Overexpression of PSY1 increases fruit skin and flesh carotenoid content and reveals associated transcription factors in apple (Malus × domestica)
Source: Front Plant Sci. 2022 Sep 15;13:967143. doi: 10.3389/fpls.2022.967143 (PMC9520574; doi:10.3389/fpls.2022.967143)
Supplement: Supplementary Table S1 — List of PCR primers used. [file Table_1.DOCX]

| Gene name | Abbreviation | Corresponding ‘GDDH’ Gene ID | Forward primer sequence (5’ – 3’) | Reverse primer sequence (5’ – 3’) |
| --- | --- | --- | --- | --- |
| Actin | Actin | MD04G1127400 | TGACCGAATGAGCAAGGAAATTACT | TACTCAGCTTTGGCAATCCACATC |
| Elongation factor 1-α | EF1-α | MD04G1011000 | CCAAGATTGACAGGAGGTCTGGAAA | GGAAGCATCTTCACCATACCTGCAT |
| Phytoene synthase | PSY1 | MD17G1133400 | GAGAAGGTGTATGAAGTGGTGCTG | TTCAAACCTTCAGTAATCCGTTCA |
| Phytoene synthase | PSY2 | MD09G1146800 | GAGAAGATGCTAGGAGAGGAAGAGT | CTTTGCCACTTGTCAGTCACCTTCC |
| Phytoene desaturase | PDS | MD04G1023800 | ACAAGACTGTACCAGGTTGTGAAC | GCTCCTTCCATTGAGGCTAAATAC |
| Zeta-carotene desaturase | ZDS1 | MD04G1220900 | CAAAGAGGTCCTCCTCCTTCTGG | GGTAGAACTCTGGTTCAGGTGGAA |
| Zeta-carotene desaturase | ZDS2 | MD12G1237300 | TCCAGTAACCGGTCGCTGTATAGT | CGTTAACCCCCATGTCAGAAAC |
| Carotenoid isomerase | CRTISO | MD14G1044400 | TCACTTGAGGAGCCAATCTACC | GCGAGCTATATCCCCAGCATT |
| Lycopene beta-cyclase | LCB1 | MD00G1049000 | GCTAGTAGCAGTGCTCTTCTGGAG | CTAGGTCCACAACAAGACCCTTTG |
| Lycopene beta-cyclase | LCB2 | MD03G1148700 | CTTGGATCCTTATTACTGGCAAGG | ATATCAAACCTAGATGGGGTGGAG |
| Lycopene epsilon-cyclase | LCE | MD02G1083500 | CGGAGCCACTATGATTAGAACCTA | AGTGACCTTCAAAAGTAGGCAGTG |
| Beta-carotene hydroxylase | BCH1 | MD07G1278900 | CTCTCTATCTCAACGCTCAACCTC | GTAGGAGGAGGAGTGGATTAGGC |
| Beta-carotene hydroxylase | BCH2 | MD01G1208300 | GTGGTCTTTGTTCACCCCAACT | ATGACGTAGGAGGACTGGATTAGG |
| Epsilon carotene hydroxylase | ECH | MD14G1097800 | GGTTCCGGATCAGAAAATTAGC | ACGAAGACGAGGATGATGTAGC |
| Zeaxanthin epoxidase | ZEP1 | MD02G1172400 | CAGATACAGAGCAACGCGTTGG | TCAACGAGGCCATTGATCCTGT |
| Zeaxanthin epoxidase | ZEP2 | MD15G1284500 | GGTTGTGTTACTGGGGATAGGA | TTCCACTGCAGGAGTGAATGTA |
| Basic helix loop helix | bHLH36 | MD09G1233000 | CTTGAAGAAGAACAGGCGGACGTTG | GCCCCAAAATCCAACACAGACTTG |
| Domain one finger | DOF2 | MD05G1018200 | GGTACTGAAGATGATCCGTCCGG | CATCAGGGGTGGTGGAGTTGTG |
| Related to AP2 | RAP2 | MD17G1152400 | GCACTGAAATCCTCTACTCCATCCG | GAGTCTTTGAGCCTTGTTCTCCCC |
| Ethylene responsive factor | ERF62 | MD04G1009000 | CACCTACTTCTCCACCTTCCTTGTC | GACGATGGGGATGATGATGATGATGG |
| Myeloblastosis transcription factor | MYB73 | MD15G1288600 | AGGGATGATAAGGAAGGAGGTGAGG | GTTCCAAAACCCCTCATTACCACCG |
| NAC transcription factor | NAC9 | MD01G1093500 | GATGAACTTTGTCCTCTGGCGC | TCCACTGGCTCTGCAATCACG |

Supplementary Table 1. List of RT-PCR primers used in the study
