# Supplementary material for: Overexpression of PSY1 increases fruit skin and flesh carotenoid content and reveals associated transcription factors in apple (Malus × domestica)
Source: Front Plant Sci. 2022 Sep 15;13:967143. doi: 10.3389/fpls.2022.967143 (PMC9520574; doi:10.3389/fpls.2022.967143)
Supplement: Supplementary Table S6 — Correlation coefficient (r) of the relationship between gene expression of carotenoid-associated TFs, apple carotenoid genes and carotenoid content in fruit skin and flesh. Color gradient from red to green, with arrows, indicate weak to strong correlations. [file Data_Sheet_1.PDF]

	PSY1	*PSY2*	*PDS*	*ZDS1*	*ZDS2*	*LCB1*	*LCB2*	*BCH1*	*BCH2*	TOTCAR
**TFs**	Fruit Skin									
MdbHLH36	0.90	0.79	0.83	0.71	0.65	0.77	0.78	0.69	0.64	0.52
MdDOF2	0.59	0.60	0.36	0.75	0.71	0.66	0.83	0.65	0.22	0.82
MdRAP2	0.84	0.75	0.65	0.88	0.83	0.87	0.97	0.82	0.51	0.82
MdERF62	0.47	0.39	0.34	0.67	0.66	0.63	0.50	0.65	0.48	0.75
MdMYB73	0.74	0.67	0.64	0.87	0.86	0.88	0.84	0.89	0.64	0.70
MdNAC9	0.83	0.74	0.76	0.90	0.86	0.92	0.82	0.90	0.77	0.76
	Fruit flesh									
MdbHLH36	0.72	0.85	0.19	0.84	0.84	0.75	-0.24	0.53	0.06	0.93
MdDOF2	0.71	0.79	0.25	0.79	0.77	0.70	-0.31	0.39	-0.07	0.93
MdRAP2	0.78	0.84	0.26	0.86	0.88	0.79	-0.13	0.55	0.04	0.90
MdERF62	0.78	0.88	0.19	0.85	0.90	0.78	-0.16	0.58	0.08	0.92
MdMYB73	0.39	0.51	0.10	0.79	0.75	0.80	0.21	0.61	0.38	0.54
MdNAC9	0.16	0.04	0.30	0.28	0.11	0.28	-0.37	-0.13	-0.33	0.34
